# Does Scoliosis-Specific Exercise Treatment in Adolescence Alter Adult Quality of Life?

**DOI:** 10.1155/2014/539671

**Published:** 2014-11-10

**Authors:** Maciej Płaszewski, Igor Cieśliński, Paweł Kowalski, Aleksandra Truszczyńska, Roman Nowobilski

**Affiliations:** ^1^Faculty of Physical Education in Biała Podlaska, Institute of Physiotherapy, Józef Piłsudski University of Physical Education in Warsaw, Akademicka 2, 21-500 Biała Podlaska, Poland; ^2^Centre for Corrective and Compensatory Gymnastics, Lompy 7, 43-300 Bielsko-Biala, Poland; ^3^Faculty of Rehabilitation, Józef Piłsudski University of Physical Education in Warsaw, Marymoncka 34, 00-968 Warsaw, Poland; ^4^Faculty of Health Sciences, Institute of Physiotherapy, Jagiellonian University, Michałowskiego 12, 31-126 Kraków, Poland

## Abstract

*Objective*. Health-related quality of life in adults, who in adolescence participated in a scoliosis-specific exercise program, was not previously studied. *Design*. Cross-sectional study, with retrospective data collection. *Material and Methods*. Homogenous groups of 68 persons (43 women) aged 30.10 (25–39) years, with mild or moderate scoliosis, and 76 (38 women) able-bodied persons, aged 30.11 (24–38) years, who 16.5 (12–26) years earlier had completed scoliosis-specific exercise or observation regimes, participated. Their respiratory characteristics did not differ from predicted values. The WHOQOL-BREF questionnaire, Oswestry Disability Questionnaire, and pain scale (VAS) were applied. *Results*. The transformed WHOQOL-BREF scores ranged from 54.6 ± 11.19 in the physical domain in the mild scoliotic subgroup to 77.1 ± 16.05 in the social domain in the able-bodied subgroup. The ODQ values did not generally exceed 5.3 ± 7.53. Inter- and intragroup differences were nonsignificant. Age, marital status, education, and gender were significantly associated with the ODQ scores. Significant association between the ODQ and WHOQOL-BREF social relationships domain scores with the participation in exercise treatment was found. *Conclusions*. Participants with the history of exercise treatment generally did not differ significantly from their peers who were only under observation. This study cannot conclude that scoliosis-specific exercise treatment in adolescence alters quality of life in adulthood.

## 1. Introduction

Adolescent idiopathic scoliosis, considered as the most predominant orthopedic condition affecting children [1–3], also prevails in adult life [4, 5] and may have lasting consequences [6–8]. Both the deformity itself (however, with a poor correlation between radiographic and patient-centered outcomes [4, 6, 9]), but also surgical and orthotic treatments, can be associated with adverse events, such as limitations in participation and intimate relationships, lower marriage rates, poor self-perception, and mental disorders [1, 5, 6, 9–12], with some authors stressing serious psychological side effects, contrasted with doubtful effectiveness of bracing [13]. Thus, scoliosis may affect one's health-related quality of life (HRQoL), defined as “the value assigned to duration of life as modified by the impairments, functional states, perceptions, and social opportunities that are influenced by injury, treatment, or policy” [14].

The concept of HRQoL “refers to the subjective evaluation of one's ability to perform usual tasks and their impact on one's everyday physical, emotional and social well-being” [15] and is a remarked issue in surgically treated [16, 17], braced, and observed [1, 5, 9] adolescent patients and their families [18]. Several long-term outcome studies addressing effects of bracing, surgery, observation (watchful waiting), and natural history studies [19, 20], on HRQol, including the Iowa [20], Ste-Justine [21], and Göteborg and Scoliosis Research Society [6, 22, 23] series, were also conducted in adults.

Surprisingly, no studies regarding this subject matter in persons treated with scoliosis-specific exercises (SSEs) have been reported. Systematic reviews addressing this issue have concentrated on technical or surrogate outcomes, typically curve angle and its progression [24–26]. Authors of the current rigorous Cochrane systematic review [27] considered quality of life as an outcome measure, but found no relevant studies.

Different SSE treatments, methods, or “schools” have been developed and widely used, especially in Europe [28, 29]. The methods remain controversial in terms of the evidence base for their effectiveness [24–27], and expert opinions are also discrepant [30, 31]. Nonetheless, therefore, such a study, especially regarding long-term effects, was needed and warranted.

We conducted a study among young adult men and women, who in adolescence participated in a specific therapeutic exercise regime.

*Our aims* were to determine the incidence and associations between curve severity, past treatment with scoliosis-specific exercises (applied in adolescence), and present functional status and HRQoL, in comparison with subjects who were diagnosed with scoliosis in adolescence but were not enrolled for the exercise treatment and were only under observation.

Spine deformity, especially in adult patients, may be associated with back pain and may potentially, depending on the severity of the deformity, lead to respiratory complications [1–3, 5]. Both issues correspond to HRQoL [32]; therefore we also studied self-reported disability due to low back pain and spirometric and total lung capacity measurements of the persons involved in the study.

## 2. Methods

We followed the recommendations of Strengthening the Reporting of Observational Studies in Epidemiology (STROBE) statement [33].

### 2.1. Study Design, Enrollment Procedure, and Participants

#### 2.1.1. Medical Records

We analyzed the medical records of 5017 children enrolled for conservative treatment or observation through a screening program for idiopathic scoliosis, conducted between 1984 and 1995 in the Centre of Corrective and Compensatory Gymnastics, Bielsko-Biala, Poland. The centre provided scoliosis screening for schoolchildren from the urban and suburban population of about 300 000 inhabitants. We excluded registries of those children in whom bracing and/or surgical treatment were recommended, and, with the use of a random numbers table, randomly selected 250 registries of the children who were enrolled for SSE treatment or observation.

#### 2.1.2. Interventions in Adolescence

The regime involved scoliosis-specific, symmetrical, strengthening, antigravity, and elongating exercises of the postural muscles. Exercises were performed in group during 45-minute gym sessions twice a week and individually at home (sets of 12–15 exercises, 30–45 minutes a day). The remaining children were under observation for three to five years on the basis of scheduled follow-up orthopedic examinations. The diagnosing orthopedic surgeon, based on physical examination and radiograph, made a decision regarding the introduction of the SSE treatment. At that time, they did not, however, follow the Scoliosis Research Society criteria for the minimal Cobb angle of 11° for scoliosis, and the so-called “scoliotic posture” was also regarded as spinal deformity.

#### 2.1.3. Enrollment Procedures

Subsequently, we attempted to locate the subjects. As, after 14–25 years, many of the potential participants changed addresses and telephone numbers; unless locating the subjects from their original addresses, we tried to retrieve the current contact data from their parents or other residents. We also applied other procedures suggested to increase participation [34]: having published an invitation letter in the city council free newspaper, provided personalized introductory letters, and made follow-up telephone calls to nonrespondents.

We managed to locate 189 (75.6% of the selected registries) potential participants. Seven addresses were not found, 49 people had emigrated, and four potential participants had died. Twenty-six people (10.4% of the initial cohort and 13.8% of the located persons) refused to participate. Fifteen people were subsequently excluded due to severe scoliosis (*n* = 6), recent X-ray exposure (*n* = 1), mental condition (*n* = 1), history of treatment of depression or other psychological disorders (*n* = 2), and noncompliance with treatment regimen (the rate of absence from exercise sessions exceeding 20%, based on patients' records) (*n* = 5). Of 149 participants finally included in the study, 2 dropped out and 3 did not return the questionnaires. Finally, a total of 144 (57.6% and 96.64% of the initially selected and finally enrolled subjects, resp.), 81 women and 63 men, completed the study. The intergroup differences in the distribution of demographic variables were nonsignificant (Table 1).

*Follow-up period* since the termination of treatment was 16.5 (12–26) years for the whole group, 17.1 (12–25) years for the exercising group, and 15.9 (12–23) years for the observation group. The included subjects' mean age at diagnosis was 10.5 (range 9–16) years. Seventy-one subjects were referred to observation, and 73 subjects started the exercise treatment. Details regarding the flow of recruitment participants, enrolment criteria, and selection process are presented in a flowchart in a separate report [35]. Below, in Table 1, we provide the demographic characteristics of the participants, who completed the study and required to interpret the results and findings of the presented study.

### 2.2. Curve Measurements

To obtain current spine deformity characteristics, two blinded specialists independently measured the magnitude of the curvature, using the Cobb method [1, 3], on a full-length anteroposterior spine radiograph. Based on their findings, we divided the participants into two groups of able-bodied (nonscoliotic) subjects and persons with mild (11–24°Cobb) or moderate (25–44°Cobb) scoliosis (Tables 1 and 2).

We enrolled subjects with mild and moderate scoliosis (*n* = 62, 92% and *n* = 6, 8% of the persons with scoliosis, resp.).  Table 2 provides more detailed characteristics of the deformity in the persons with scoliosis.

### 2.3. Pulmonary Function

Spirometry and body plethysmography (total lung capacity, TLC) measurements were conducted by highly trained technicians in the Laboratory of the Centre of Pulmonology and Thoracic Surgery, Bystra, Poland, in accordance with the standard European Respiratory Society's (ERS) formula, using Lungtest 1000 spirometer, MES, Poland, and Bodyscreen system body plethysmograph, Jaeger, Germany. Body plethysmography and spirometric measurements were taken in subjects in a sitting and in a standing position, respectively.  Table 3 includes respiratory characteristics of the subjects, with intergroup comparisons. The values are expressed as percentages of the predicted values (ERS norms).

### 2.4. Outcome Measures

#### 2.4.1. HRQoL

We used the WHO Quality of Life-BREF (WHOQOL-BREF) questionnaire. It comprises 26 items, which measure the following broad domains: physical health, psychological health, social relationships, and functioning in environment. The WHOQOL-BREF is a shorter version of the original instrument (WHOQOL100). The questionnaires were designed to assess the individual's perceptions in the context of their culture and value systems and their personal goals, standards, and concerns [36, 37]. We applied the Polish version of the WHOQOL-BREF instrument [38].

#### 2.4.2. Disability

To measure the subjects' permanent disability associated with low back pain, we used the revised Oswestry Disability Index (also known as the Oswestry Low Back Pain Disability Questionnaire (ODQ)) [39, 40]. The ODQ is applied to measure activity limitation in the participants due to low back problems and comprises ten sections: pain, personal care, lifting, walking, sitting, standing, sleeping, sexual life, social life, and travelling [40, 41]. The ODQ is considered the “gold standard” of low back functional outcome tools [41] and, as stressed by its developers [40], was designed to measure physical disability rather than impairment. We used the validated Polish version of the instrument [42].

#### 2.4.3. Pain

The participants reported back pain severity on a Visual-Analogue Scale, on five-day recall basis, labelled from “no pain” to “maximal pain I can imagine” on a 100 mm line.

### 2.5. Statistics

We used descriptive statistics for the demographic and clinical characteristics of the subjects. To assess intergroup differences for subsequent characteristics and individual ODQ severity ranges, we applied the maximum-likelihood chi-square test. The differences regarding subsequent demographic and clinical characteristics in relation to the pain severity (VAS scale) scores were computed with the median *U*-test. To examine the interaction between the ODQ scores and a number of confounders, we employed the regression models zeroinfl()/hurdle(), from the PSCL package [43, 44]. We chose that model as the regression model based on the Poisson distribution appeared worse than the zero inflated model, which we had verified with the Vuong test, comparing these two models (Table 7). The WHOQOL-BREF raw scores were first transformed to the 0–100 values within subsequent domains. Then, we used the MASS package to calculate the interactions between the WHOQOL-BREF domain scores and the individual confounders, with the use of the backward elimination regression method [45]. Data were analyzed using R version 2.14.0.

## 3. Results

**Figure 1:**
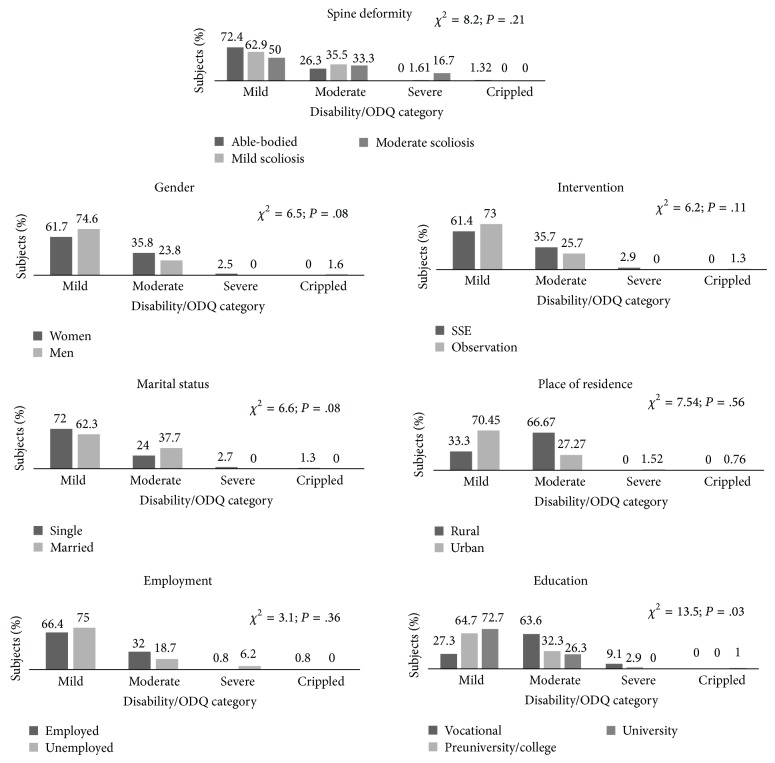
Low back functional disability as presented within the ODQ categories, related to subsequent variables. ODQ categories: 0–20% minimal; 21–40% moderate; 41–60% severe disability; 61–80% crippled; 81–100% bed-bound or patients exaggerating their symptoms.

The subsequent WHOQOL-BREF domains, in scores transformed to 0–100 values, ranged from 54.6 ± 11.19 in the physical health domain in the mild scoliotic subgroup to 77.1 ± 16.05 in the social relationship domain in the able-bodied subgroup. The differences were nonsignificant, both between groups and between mild and moderate scoliotics. For there are no Polish norms, we were unable to compare those findings with reference norms. The low values of ODQ in the majority of the subjects, not exceeding 5.3 ± 7.53 and with nonsignificant differences between groups, indicate minor disabilities, caused by low back pain [39, 41]. Detailed data, with inter- and intragroup comparisons, are presented in Table 5. Additionally, we present in Figure 1 results of the analyses regarding individual ODQ categories, in relation to subsequent variables.

The majority of the participants, both able-bodied and with AIS, regardless the magnitude of the deformity, did not report severe back pain, with the greatest VAS scores not exceeding 18.82 ± 14.57 (in the 0–100 scale), found in persons with vocational educational background. However, individual participants with moderate scoliosis reported pain levels exceeding 50. We tested the obtained VAS scores against a number of variables, including presence of spine deformity and type of therapeutic intervention, and found no significant differences (Table 6).

Tables 7 and 8 provide data from the multiple regression analyses for ODQ and WHOQOL-BREF, with the subsequent domains analyzed. As confounding factors, which may have been associated with the obtained results, we included age, gender, presence of scoliosis (>10°Cobb), intervention (SSEs versus observation), marital status, employment, and level of education. Results are shown in Tables 7 and 8, respectively. For WHOQOL-BREF we report findings with significant or nearly significant differences (Table 8), obtained using the regression backward elimination model. The remaining variables did not influence the results.

As we anticipated, variables that could be linked with the general health and well-being (age, marital status, education, and gender) appeared to be significantly associated with the ODQ scores (Table 7). Also, we found a significant association of both the ODQ and WHOQOL-BREF social relationships domain scores with the participation in SSE regime (Tables 7 and 8).

## 4. Discussion

### 4.1. Demographic and Clinical Characteristics

#### 4.1.1. Demographics

The study involved adult participants, divided into subgroups of able-bodied (Cobb angle ≤10°) and scoliotic subjects, with a history of either SSE treatment,or observation for adolescent scoliosis. The intergroup differences as regards demographic characteristics were nonsignificant (Table 1). Therefore, we assumed that multiple regression analyses and analyses of variance, considering demographical and clinical characteristics, and interventions, as factors confounding current self-reported HRQoL, disability due to low back pain, and pain severity, were allowed. We agree with Wang et al. [46] who postulated incorporating geographical factors in HRQoL analyses for patients with AIS. Therefore, we included the place of residence (urban versus rural) in our analyses. We considered educational level, employment, and marital status, as current factors potentially influencing the analysed person-reported outcomes, while many other reports, as recently systematically reviewed by Rushton and Grevitt [17, 19], concentrated on condition-specific characteristics, for example, curve magnitude or severity of trunk deformation.

#### 4.1.2. Respiratory Function

Restrictions in lung volumes, as measured with body plethysmography (TLC), but also estimated with more accessible spirometric measurements (FVC) [47], are associated with severe structural scoliosis [47, 48]. However, reduction in FVC was also reported in patients with deformities smaller than 35°Cobb [47]. Therefore, we measured total lung capacities and performed spirometric measurements in the subjects. The results show that neither the scoliotic nor the able-bodied participants manifested reductions in lung capacities or restrictive lung defects. Participants with moderate scoliosis did not differ significantly from their peers with mild deformities (Table 3). Therefore, we assumed that lung capacity and respiratory function were not restricted in the participants; thus we ruled out this potential confounding factor and did not consider it in further analyses. We refrained from further analyses, as we found no restrictions in expiratory flow rates (FVC_ex_), while FEV_1_/FVC_ex_ ratio is considered normal; even lung volumes are restricted [47]. As smoking might have been influencing the respiratory parameters, we also analysed this variable. We found no significant associations (Table 4); however we considered smoking as a dichotomous variable and did not perform any more detailed analyses, for example, number of cigarettes a day, or pack-years of smoking analyses. Nonetheless, our observations were in concert with findings in adults 25 years after brace or surgical treatment, in whom smoking habits and curve size were shown to be no risk factors for reduced pulmonary function [49]. We cannot formulate any firm conclusions from these measurements, but our observations correspond with the physiotherapy guidelines for patients with scoliosis, encouraging any forms of physical activity [50], especially in view of the evidence that scoliosis surgery should not be used to increase lung vital capacity as no such effects of surgery have been confirmed [51].

### 4.2. Outcomes

*Quality of life* is related to health, both mental and physical, and incorporates aspects of objective physical functioning and subjective sense of well-being [52]. This description reflects the general objective of our study. We aimed to assess functioning of persons who in adolescence were engaged in the scoliosis-specific exercise programme or were observed rather than concentrate on an evaluation of the effectiveness of a particular procedure or on a detailed analysis of the deformity within the biomechanical frame of reference.

There are many generic, condition-specific, and even superspecific measures of self-reported HRQoL and body image of patients with scoliosis developed [53]. We utilised the WHOQoL-BREF, a generic measure of the subjectively perceived impact of a disease, and its treatment on physical, mental, social, and environmental dimensions of HRQoL [36], rather than a condition-specific tool. One of the condition-specific SRS questionnaires has been adapted for adult populations [54], but the one available in Polish version [55] was developed and validated for use in adolescent populations of patients treated surgically [53] or as an outcome instrument of brace treatment [55]. Moreover, as our study regarded able-bodied persons and persons with mild-to-moderate deformities, our aim was to investigate their general HRQoL. Also, we were interested in how the treatment procedures, not only the condition itself, may have influenced HRQoL. The WHOQoL-BREF and another widely known generic HRQoL measurement instrument, SF-36 (e.g. [6]), were also used by other authors investigating long-term HRQoL outcomes of adult persons, nonsurgically treated for AIS [56].

Haefeli et al. [56] included in their study, among patients treated with a brace, adult persons who in adolescence had undergone physiotherapy treatment. The authors found no differences in general WHOQoL-BREF between nonsurgically treated and control groups. In contrast with our study, they did not, however, investigate the associations of the different treatment modalities with the WHOQoL-BREF scores. The physiotherapy methods are not described; we doubt whether those were condition-specific exercises. The authors found no significant differences in the disability levels measured with ODQ with the WHOQoL-BREF physical domain. The study involved patients with more severe deformities than in our study (≥45°Cobb). Those patients reported more pain than participants with milder curves. We also found no significant differences between able-bodied and scoliotic participants, regardless the severity of the deformity (Table 3). Our study, however, involved mainly persons with mild scoliosis and only six participants with moderate deformities not exceeding 40°Cobb.

Despite considerably large body of evidence regarding HRQoL and subjective functioning of adults with AIS [4, 5, 9], we were unable to compare our findings with any other studies addressing adults with a history of scoliosis-specific exercise treatment for AIS. We have recently conducted a comprehensive overview of systematic reviews addressing any conservative treatments of AIS (protocol registered at PROSPERO, CRD York, CRD42013003538, full text in press), and have found only one systematic review analysing HRQoL as an outcome. Other papers either considered surrogate outcomes (e.g., curve severity, trunk rotation) [24–26] or failed to find relevant studies in terms of study design and/or methodological rigour [27]. The systematic review, by Davies et al. [57], regarded bracing and other nonsurgical interventions and found limited evidence suggesting that bracing may negatively influence quality of life. Remarkably, Davies et al. concluded that it was not known whether bracing was more effective in reducing curve progression than Side-Shift therapy (a method of SSE) [57].

For those reasons, the presented paper reports findings of the first investigation regarding this subject matter and was believed to enhance the body of knowledge in this subject. We refrained from discussing findings of other authors, as they do not correspond directly with our study and have been broadly discussed by other authors [1–5, 9] and also in our previous report [35].

#### 4.2.1. Limitations of the Study

We present data obtained from an uncontrolled observational study with a long follow-up of 16.5 years, a comparatively low level of evidence study design [58], prone to bias [59]. As we described in Section 2, we followed a number of procedures to reduce selection bias [34]. Participants did not differ significantly in demographic (Table 1) and clinical (Tables 2–4) characteristics. Thus, we assume that these findings are not limited to the studied population. Nonetheless, in the sample of 144 participants, with only about 3% finally enrolled subjects who were lost to follow-up, only 57.6% of the initially selected persons agreed to participate, a number below a threshold response rate proposed for a rigorous observational study [59]. It is difficult to determine whether the enrolment procedure might have caused some selection bias, for example, by encouraging better educated persons for participation. Furthermore, able-bodied subjects may believe that the study does not really benefit them and hence may be less likely to participate. Also, in some instances, the invitation was passed to the potential participants via their relatives (most frequently parents). Thus, a potential selection bias may have occurred.

In conclusion, self-perceived health-related quality of life and disability due to low back pain in adult persons who in adolescence took part in an intensive scoliosis-specific exercise programme did not differ significantly from their peers who were only under observation due to scoliosis. Nonetheless, treatment undergone in adolescence, but also current factors, such as employment, marital status, and education, were associated with self-perceived quality of life and levels of physical disability corresponding with back pain. This study did not contribute to the body of evidence as regards effectiveness of scoliosis-specific exercise programmes, but in our opinion, may enhance the body of knowledge as regards possible lasting side effects of these interventions. Further studies, possibly with longer follow-up period, are necessary to better explore this subject matter.

## Figures and Tables

**Table 1:** Characteristics of the participants and intergroup comparisons.

Factor or domain	Total group (*n* = 144)	Able-bodied (*n* = 76)	Scoliotics (*n* = 68)	*P*
Age	30.11 ± 4.11	30.11 ± 3.99	30.10 ± 4.67	.98
	(24–39), 30	(24–38), 30	(25–39), 30	
Women (*n*)	81	38	43	.10
Place of residence (*n*)				
Rural	8	5	3	.18
Urban ≤ 20 000	1	0	1	
Urban 20 000–50 000	2	0	2	
Urban > 50 000	133	71	62	
Marital status (*n*)				
Single	75	35	40	.17
Married/living together	69	41	28	
Education (*n*)				
Vocational	11	5	6	.88
Preuniversity/college	34	18	16	
University	99	53	46	
Intervention (*n*)				
Observation	73	41	32	.37
Exercises	71	35	36	

Data for age are presented as mean ± SD (range), median.

**Table 2:** Clinical characteristics of the participants with scoliosis.

Curve size (°Cobb) (*n* = 68)	Curve severity (*n*, (%))		Curve location (*n*, (%))			Scoliosis type (*n*, (%))	
	11–24°Cobb (mild)^*^	25–40°Cobb (moderate)^*^	Single primary Th	Single primary Th-L or L	Double major	Early-onset idiopathic^**^	Adolescent idiopathic^***^
15.16 ± 6.44 (11–36)	62 (92)	6 (8)	9 (13)	45 (66)	14 (25)	11 (16)	57 (74)

Curve size expressed as mean ± SD (range); ^*^Scoliosis Research Society classification; ^**^9 years of age; ^***^10–16 years of age; Th: thoracic; L: lumbar.

**Table 3:** Lung volumes and respiratory function of the participants.

	Total group (*n* = 141^*^)	Able-bodied (*n* = 75^*^)	Scoliotics (*n* = 66^*^)	*P*	Scoliotics (*n* = 66^*^)		*P*
					Mild (*n* = 60)	Moderate (*n* = 6)	
TLC %	108.51 ± 12.8	110.39 ± 11.67	106.42 ± 13.72	.06	106.36 ± 13.29	107.00 ± 19.07	.91
VC %	106.33 ± 13.44	108.03 ± 12.51	104.43 ± 14.26	.11	104.15 ± 13.53	107.33 ± 27.86	.61
FVC_ex_ %	109.00 ± 12.58	109.43 ± 12.46	108.53 ± 12.80	.66	108.00 ± 12.37	114.00 ± 16.88	.28
FVC_in_ %	98.65 ± 19.21	101.24 ± 19.42	95.79 ± 18.71	.09	95.95 ± 20.93	94.17 ± 19.22	.83

Data are presented as mean ± SD; %: percentage of the European Respiratory Society's predicted value; TLC: total lung capacity; VC: vital capacity; FVC_ex_: forced vital capacity in exertion; FVC_in_: forced vital capacity in insertion; A: actual value in litres; ^*^three subjects (one able-bodied and two mild scoliotics, 2.8% of the whole group) were unable to perform three measurements within acceptable repeatability; thus data for 141 subjects are presented.

**Table 4:** Respiratory function of smokers and nonsmokers.

	Not smoking (*n* = 85^*^)	Smoking (*n* = 56^*^)	*P*
TLC %	108.92 ± 13.09	109.20 ± 11.78	.91
VC %	107.65 ± 15.74	106.80 ± 10.42	.75
FVC_ex_ %	109.55 ± 13.66	109.20 ± 11.61	.89
FVC_in_ %	96.50 ± 20.93	95.78 ± 19.22	.85

^*^141 completed the spirometric and TLC measurements; 3 subjects were unable to correctly follow the procedures (abbreviations are explained in Table 3).

**Table 5:** ODQ and WHOQOL-BREF scores-intergroup and intragroup comparisons.

Measures and domains	Total group (*n* = 144)	Able-bodied (*n* = 76)	Scoliotics (*n* = 68)	*P*	Scoliotics		*P*
					11–24°Cobb (*n* = 62)	25–40°Cobb (*n* = 6)	
ODQ total score	3.8 ± 4.45; 3	3.4 ± 4.56; 2	4.2 ± 4.32; 3	.25	4.1 ± 3.97; 3	5.3 ± 7.53; 3.5	.50
WHOQOLBREF domains							
Physical health	55.0 ± 9.74; 53.6	55.2 ± 8.34; 53.6	54.8 ± 11.17; 57.1	.83	54.6 ± 11.19; 53.6	57.14 ± 11.74; 58.9	.60
Psychological	66.8 ± 11.61; 66.7	66.9 ± 11.16; 70.8	66.7 ± 12.17; 66.7	.91	66.6 ± 12.45; 66.7	67.4 ± 9.65; 66.7	.88
Social relationships	74.6 ± 18.58; 75	77.1 ± 16.05; 75	71.8 ± 20.82; 75	.21	71.8 ± 20.82; 75	72.2 ± 12.55; 75	.96
Environment	61.3 ± 14.68; 62.5	62.2 ± 11.91; 62.5	60.4 ± 17.32; 62.5	.47	60.4 ± 17.25; 62.5	60.4 ± 19.73; 65.6	.99

Data are presented as mean ± SD; median.

**Table 6:** Self-reported pain severity (VAS scores) as related to different independent variables.

Independent variables	Pain severity [VAS score, millimeters]	*P*
Gender		
Males	12.49 ± 18.51; 5	.39
Females	15.10 ± 17.92; 9	
Place of residence		
Rural	17.67 ± 15.81; 7	.52
Urban	13.62 ± 18.38; 5.5	
Scoliosis		
Able-bodied	14.36 ± 18.10; 10	.82
Mild	13.13 ± 17.62; 5	
Moderate	17.50 ± 26.50; 4.50	
Intervention		
Exercise treatment	16.86 ± 19.96; 9.5	.06
Observation	11.22 ± 15.94; 4	
Marital status		
Single	13.11 ± 19.61; 3	.56
Married	14.88 ± 16.53; 10	
Education		
Vocational	18.82 ± 14.57; 8	.13
Preuniversity/college	18.41 ± 20.66; 14	
University	11.89 ± 17.38; 4	
Employment		
Employed	8.19 ± 9.87; 3	.18
Unemployed	14.68 ± 18.85; 7.5	

Data are presented as mean ± SD; median; VAS score range: 0–100.

**(a):** 

Total ODQ score	Parameter estimate	Standard error	*P*	Odds ratio	95% CI
Age (older)	.033	.014	.02^*^	.01	.01–.06
Gender (male)	−.003	.097	.98	.009	.02–.19
Place of residence (rural)	−.088	.054	.11	.009	.001–.02
Scoliosis (≤10°Cobb)	.140	.091	.13	.01	.03–.32
Intervention (observation)	−.024	.093	.80	.009	.01–.16
Marital status (single)	−.213	.096	.03^*^	.008	.001–.03
Employment (employed)	−.128	.152	.40	.008	.004–.17
Education (lower level)	−.131	.065	.04^*^	.008	.002-.003

**(b):** 

Total ODQ score	Parameter estimate	Standard error	*P*	Odds ratio	95% CI
Age (older)	.103	.071	.15	.01	.03–.24
Gender (male)	.893	.447	.04^*^	.02	.01–1.77
Place of residence (rural)	.289	.417	.49	.01	.2–1.10
Scoliosis (<10°Cobb)	−.028	.434	.95	.009	.8–1.83
Intervention (observation)	1.177	.483	.01^*^	.03	.1–2.12
Marital status (single)	−1.207	.491	.01^*^	.02	.02–.24
Employment (unemployed)	.487	.687	.48	.01	.8–1.83
Education (lower level)	.850	.420	.04^*^	.02	.2–1.67

^*^Difference significant,  *P* < .05; likelihood ratio: *P* < .01.

**Table 8:** Multiple regression analysis for WHOQOL-BREF social relationships domain scores: the backward elimination model.

Variable	Parameter estimate	Standard error	*P*
Intervention (observation)	5.15	3.03	.09
Employment (employed)	−12.49	4.82	.01^*^

^*^Difference significant,  *P* < .05; likelihood ratio: *P* < .05; McFadden *R* = .05.
